# Linking microbial co‐occurrences to soil ecological processes across a woodland‐grassland ecotone

**DOI:** 10.1002/ece3.4346

**Published:** 2018-07-22

**Authors:** Samiran Banerjee, Peter H. Thrall, Andrew Bissett, Marcel G. A. van der Heijden, Alan E. Richardson

**Affiliations:** ^1^ CSIRO Agriculture and Food Canberra ACT Australia; ^2^ Agroscope, Research Division Agroecology and Environment Plant‐Soil‐Interactions Group, Reckenholz Zurich Switzerland; ^3^ CSIRO Ocean and Atmosphere Hobart Tas Australia; ^4^ Department of Evolutionary Biology and Environmental Studies University of Zürich Zürich Switzerland; ^5^ Institute of Environmental Biology Faculty of Science Utrecht University Utrecht The Netherlands

**Keywords:** ammonia oxidizers, ecotone, extracellular enzymes, keystone taxa, kriging, microbial networks

## Abstract

Ecotones between distinct ecosystems have been the focus of many studies as they offer valuable insights into key drivers of community structure and ecological processes that underpin function. While previous studies have examined a wide range of above‐ground parameters in ecotones, soil microbial communities have received little attention. Here we investigated spatial patterns, composition, and co‐occurrences of archaea, bacteria, and fungi, and their relationships with soil ecological processes across a woodland‐grassland ecotone. Geostatistical kriging and network analysis revealed that the community structure and spatial patterns of soil microbiota varied considerably between three habitat components across the ecotone. Woodland samples had significantly higher diversity of archaea while the grassland samples had significantly higher diversity of bacteria. Microbial co‐occurrences reflected differences in soil properties and ecological processes. While microbial networks were dominated by bacterial nodes, different ecological processes were linked to specific microbial guilds. For example, soil phosphorus and phosphatase activity formed the largest clusters in their respective networks, and two lignolytic enzymes formed joined clusters. Bacterial ammonia oxidizers were dominant over archaeal oxidizers and showed a significant association (*p* < 0.001) with potential nitrification (PNR), with the PNR subnetwork being dominated by *Betaproteobacteria*. The top ten keystone taxa comprised six bacterial and four fungal OTUs, with Random Forest Analysis revealing soil carbon and nitrogen as the determinants of the abundance of keystone taxa. Our results highlight the importance of assessing interkingdom associations in soil microbial networks. Overall, this study shows how ecotones can be used as a model to delineate microbial structural patterns and ecological processes across adjoining land‐uses within a landscape.

## 1 INTRODUCTION

Ecotones between adjacent ecosystems or biomes that harbor contrasting plant communities represent useful areas for investigation, as they support unique ecological dynamics (Anadón, Sala, & Maestre, 2014; Archer & Predick, 2014). However, recent studies show that ecotones are highly responsive to environmental change and this is especially true for ecotones in the arid and semi‐arid regions such as the ones in Australia (Delgado‐Baquerizo et al., 2014). Grassland‐woodland ecotones around the world are subject to dynamic shifts toward an unstable state, and this has received considerable research attention in recent years (Bradford, Schlaepfer, Lauenroth, & Burke, 2014; Sala & Maestre, 2014). Ecotones encompass interactions occurring between adjoining systems and are useful because the local effects of shifts in vegetation can be explicitly assessed independently of the environmental variability that may occur over broader spatial scales (Gosz, 1993). In addition, such areas can reveal the edge effect between two adjacent habitats (Lacasella, Gratton, & De Felici, 2015; Malmivaara‐Lämsä et al., 2008; Murcia, 1995). Edge effect is the result of the abiotic and biotic interactions between adjoining habitats when the habitats are separated by an abrupt transition (sensu Murcia, 1995).

Previous studies have focused on ecotones to examine impacts on community structure (e.g., species diversity and distribution patterns) as well as a range of ecological processes such as above‐ground biotic interactions, hydrology, fire dynamics, and responses to climate change (Archer & Predick, 2014; Eldridge et al., 2011; Ratajczak, Nippert, Briggs, & Blair, 2014). In contrast, understanding of belowground communities and interactions within the soil microbiota has received less attention (Malmivaara‐Lämsä et al., 2008). Soil microbiota provide a range of important ecosystem services including soil aggregation, organic matter decomposition, nutrient cycling, and mutualistic and pathogenic interactions with plants (Bardgett & van der Putten, 2014; Killham, 1990; Richardson, Barea, McNeill, & Prigent‐Combaret, 2009; Schimel & Schaeffer, 2012; van der Heijden, Bardgett, & Van Straalen, 2008). While patterns across ecotones have been observed for some soil parameters (e.g., moisture, temperature, carbon storage, etc.) and macrobiota in previous studies (Lacasella et al., 2015; Magura, 2017; Schmidt, Jochheim, Kersebaum, & Lischeid, 2017), little information is available on soil microbiome (Malmivaara‐Lämsä et al., 2008).

The soil microbiome comprises a vast diversity and abundance of different microbial groups and complex trophic interactions (Bardgett & van der Putten, 2014; van der Heijden et al., 2008). Microbial co‐occurrence networks can reveal associations among network members and yield insight into microbiome functioning (Bissett, Brown, Siciliano, & Thrall, 2013; Cardona, Weisenhorn, Henry, & Gilbert, 2016; Faust & Raes 2012; Fuhrman, 2009;). For example, patterns of microbial co‐occurrence have been demonstrated for a diverse range of aquatic and terrestrial environments (Banerjee, Baah‐Acheamfour et al., 2016; Barberán, Bates, Casamayor, & Fierer, 2012; De Menezes et al., 2015; Graham et al., 2017; Shi et al., 2016). Previous studies using network analysis have often only assessed bacterial communities and not fungal or archaeal communities (Banerjee, Baah‐Acheamfour et al., 2016; Barberán et al., 2012; Shi et al., 2016; Vick‐Majors, Priscu, & Amaral‐Zettler, 2014). Thus, the roles of these latter groups have been underrepresented in microbial network analyses and only a few studies have investigated associations all three kingdoms (Ma et al., 2016; Steele et al., 2011). Moreover, network analysis provides a statistical tool to identify keystone taxa that play a key role in microbiome structure and functioning (Banerjee, Schlaeppi, & van der Heijden, 2018; Power et al., 1996). A number of studies have used network‐based scores to identify putative keystone taxa in different environments (Hartman et al., 2018; Lupatini et al., 2014; Shi et al., 2016; Vick‐Majors et al., 2014) and linked their abundance to soil nutrient cycling processes (Banerjee, Kirkby et al., 2016; Li, Chen, Zhang, Yin, & Huang, 2017).

A major challenge in ecology is to link microbial co‐occurrences to processes that contribute to soil function. For example, extracellular enzymes are ubiquitous in soil environments and play critical roles in ecosystem functioning through mediation of carbon (C), nitrogen (N), and phosphorus (P) mineralization, thus, facilitating soil organic matter decomposition (Burns, 1982). Soil enzyme activities have often been used as indicators of soil health and microbial function (Allison & Vitousek, 2005; Saiya‐Cork, Sinsabaugh, & Zak, 2002; Sistla & Schimel, 2013). Likewise, ammonia oxidation is important for soil nutrient availability as it is a key step for nitrification in which ammonia is converted to hydroxyl amine and subsequently to nitrite and nitrate (Kowalchuk & Stephen, 2001). The functional gene, *amoA,* is present in both bacteria and archaea and has been used in many studies to quantify the abundance of ammonia oxidizers in different environments (Di et al., 2009; Jia & Conrad, 2009; Leininger et al., 2006). Spatial patterns of ammonia oxidizers across ecotones can unravel niche differentiation and partitioning among bacteria and archaea based on nutrient availability (Prosser & Nicol, 2012). However, few studies have assessed microbial co‐occurrences in relation to soil nitrification and enzyme activities.

In a previous study, we found similar spatial patterns for a wide range of soil properties and extracellular enzyme activities across two native woodland‐grassland ecotones (Banerjee, Bora, Thrall, & Richardson, 2016). In this study, we further investigated patterns of abundance, diversity, and co‐occurrence for archaeal, bacterial, and fungal communities in one of these ecotones. Our overall hypothesis was that soil microbial properties are different in the transition zone than either of the adjacent woodland or grassland communities. A multifarious approach was used to address the following specific questions: (a) How do the spatial structure, composition, and co‐occurrences of soil archaeal, bacterial, and fungal communities change across a woodland‐grassland ecotone?; (b) How are ammonia oxidizing bacteria and archaea linked to potential nitrification across such ecotones?; (c) Is the composition of microbial networks related to soil properties and ecological processes?; and (d) Which soil properties drive the abundance of microbial keystone taxa across the woodland‐grassland ecotone?

## 2 MATERIALS AND METHODS

### 2.1 Study site and soil sampling

The study was conducted at a native woodland adjacent to native grassland within Namadgi National Park (35.6667° S, 148.950° E) in the Australian Capital Territory. The woodland was dominated by *Eucalyptus* spp. with scattered *Acacia dealbata* and *Acacia implexa* in the understorey (Banerjee, Bora et al., 2016). The grassland consisted of a mix of native grasses including *Austrodanthonia* sp. and *Themeda* sp. The mean annual rainfall at Namadgi National Park is 777.3 mm with 4.4°C and 17.3°C minimum and maximum mean annual daily temperature (http://www.weatherzone.com.au). The site was dominated by Brown Sodosols; these are typically composed of fine sandy clay loams with 10%–20% clay content (Isbell, 2002). A 50 m length × 20 m width sampling plot was established across the woodland and grassland (i.e., extending 25 m into both the woodland and grassland; Supporting Information Figure S1). A rectangular grid design consisting of 55 nodes was employed, with adjacent nodes separated by a linear or perpendicular distance of 5 m. Soil samples were collected in September 2013 by sampling at each node. Each sample consisted of a composite of 10 individual soil cores (4 cm diameter) collected at 0–10‐cm depth within a 10‐cm radius of each sampling node. The corer was cleaned between nodes and soil samples were placed on ice in a cooler box for transfer to the laboratory. Samples were processed and subsampled on the same day as collection by removing plant materials, homogenizing, and passing through a 2‐mm sieve.

For vegetation type comparisons, the experimental grid was divided into three components: woodland, transition, and grassland (20, 10, and 20 m, respectively; Supporting Information Figure S1). This assignment was based on field observations that the first 20 m (from the plot perimeters) was consistently woodland and visually homogeneous. Similarly, the last 20 m (toward the end of the sampling plot) was consistently grassland and visually homogeneous. The visual assignment of these components was substantiated with data from soil analyses and extracellular enzyme activities (Banerjee, Bora et al., 2016).

### 2.2 Soil analyses and quantitative PCR

Detailed analyses of soil properties and extracellular enzyme activities of all sample points across the ecotone were described previously by Banerjee, Bora et al. (2016). Briefly, gravimetric soil moisture content, pH, dissolved organic carbon (DOC), dissolved organic nitrogen (DON), ammonium (NH_4_), nitrate (NO_3_), percentage of total carbon (%C), and nitrogen (%N), total, inorganic, and organic phosphorus (P) were measured for each soil sample. Activities of extracellular enzymes such as cellulolytic (*β‐*1,4‐glucosidase and cellobiohydrolase), lignolytic (peroxidase and phenol oxidase), and complex N or P depolymerizing (acid phosphatase and chitinase) were determined and expressed in units of nmol hr^−1^ g^−1^. Potential nitrification activity (PNR) was measured according to Hart, Stark, Davidson, and Firestone (1994). Briefly, 10 g of sieved, field‐moist soil was mixed with 100 ml of solution containing 0.2 M monopotassium dihydrogen phosphate, 0.2 M dipotassium hydrogen phosphate, and 50 mM ammonium sulfate. The mixture was shaken at 100 rpm at room temperature for 24 hr. An aliquot of 10 ml was collected and nitrite (NO_2_), and NO_3_ were measured by colorimetric assay and spectrophotometer. The 24‐hr time point was selected based on a time course assay, and final values were calculated by comparing with time zero samples.

For all 55 soil samples, DNA was extracted from 0.25 g soil using the PowerSoil DNA isolation kit (MoBio, Carlsbad, CA) following manufacturer's instructions. DNA concentrations were determined by NanoDrop spectrophotometry (NanoDrop, Wilminghton, DE). The abundances of bacterial, archaeal, and fungal genes were determined by quantitative real‐time PCR using the Qiagen QuantiTect^™^ SYBR^®^ Green PCR Master Mix (Qiagen Inc., Victoria, Australia), a Cavro^®^ Omni Robot (Tecan Group Ltd., Seestrasse, Switzerland), and an ABI 7900 real‐time PCR machine (Applied Biosystems, Foster City, CA). Bacterial 16S rRNA and *amoA* genes were quantified using 338F and 518R (Lane et al., 1985; Muyzer, de Wall, & Uitterlinden, 1993) and amoA 1F and amoA 2R (Rotthauwe, Witzel, & Liesack, 1997) primers, respectively. Archaeal 16S rRNA and *amoA* genes were quantified using 771F and 957R (Ochsenreiter, Selezi, Quaiser, Bonch‐Osmolovskaya, & Schleper, 2003) and Arch‐amoA 104F and Arch‐amoA 616R (Alves et al., 2013) primers, respectively. Fungal ITS was quantified using the primer set ITS1F and ITS4 as reported by White, Bruns, Lee, and Taylor (1990) and Gardes and Bruns (1993). Details of thermal cycling conditions and quality assessment are provided in *Supporting Information*.

### 2.3 Amplicon sequencing

Sequencing was carried out using an *Illumina MiSeq* following a previously published protocol (Bissett et al., 2016). For sequencing, samples were randomly selected equally from three zones with six samples in each habitat component (Supporting Information Figure S1). Of the 11 rows in the sampling grid, nine rows (three for each of woodland, transition, and grassland) were selected for selection of samples, and two rows (one between two adjacent components) were selected as a buffer. Amplicons targeting the bacterial 16S rRNA gene, archaeal 16S rRNA gene, and fungal ITS genes were prepared using the 27F–519R (Lane et al., 1985; Muyzer et al., 1993), A2F–519R (DeLong, 1992) and ITS1F–ITS4 (Gardes & Bruns, 1993; White et al., 1990) primer sets, respectively. For all amplicons, Illumina 300 bp paired‐end sequencing was performed at the Australian Genome Research Facility (Melbourne, Australia). For bacterial and archaeal16S rRNA genes, the quality of R1 and R2 reads was determined using FastQC (Andrews, 2010). For fungal ITS, only R1 sequences were used that assessed the ITS1 region (Bissett et al., 2016). Reads were trimmed to remove base pairs from the end of reads after read quality per sample declined (10 and 50 bp for read1 and read2, respectively). Reads were trimmed by as many base pairs as possible while still leaving an overlap for reliable merge of R1 and R2 reads and then merged using FLASH (Magoč & Salzberg, 2011). FASTA format sequences were extracted from FASTQ files, and sequences <400 bp, with homopolymer >8 bp or containing ambiguous bases were removed using the Mothur (V1.38.0) (Schloss et al., 2009). Singletons were removed, and OTUs were defined by clustering at 97% similarity with the “cluster_otus” function in UPARSE (Edgar, 2013). Sequences were then mapped to these OTUs to produce a OTU abundance table using the “usearch_global” function in USEARCH (Edgar, 2010) and classified according to SILVA v102 using the Naïve Bayesian classifier as implemented in Mothur (Wang, Garrity, Tiedje, & Cole, 2007). For fungal ITS1 region sequences, FASTA files were extracted from FASTQ files, and complete ITS1 regions were extracted from R1 reads using ITSx (Bengtsson‐Palme et al., 2013). Partial ITS1 sequences and those not containing ITS1 were discarded. Remaining ITS1 sequences were used for OTU picking and OTU table production using the same methods described above for 16S rRNA genes. OTUs were classified as above against the ITS fungal database UNITE (V6) (Kõljalg et al., 2005). The total number of OTUs in each sample varied from 825 to 1,832 for bacteria, 15–214 for archaea, and 222–538 for fungi (Supporting Information Figure S2).

### 2.4 Statistical analyses

Alpha diversity indices such as species richness, Pielou's evenness, and Shannon‐Weaver diversity were calculated from bacterial, archaeal, and fungal OTU tables using the *vegan* package (Oksanen et al., 2017) in R v3.4 (R Development Core Team, 2016). Microbial beta diversity patterns were assessed on square‐root transformed data with principal coordinate analysis (PCoA) using Bray–Curtis dissimilarity matrix in PRIMER‐E (PRIMER‐E, Plymouth, UK). The effect of habitat edge on microbial communities was assessed by performing PERMANOVA with 999 permutations. Spatial variability was determined using geostatistical analyses in GS+ version 10 (Gamma Design Software, Plainwell, MI, USA). Spatial dissimilarity was computed by calculating the isotopic semivariance (Goovaerts, 1998). Semivariograms were calculated with a minimum of 30 sample pairs per lag class (Journel & Huijbregts, 1978). Spatial dependency (SPD) was calculated as SPD = C/(C + C_0_), where C is the structural variance, C_0_ is the nugget, and C + C_0_ is the sill. Values of SPD vary from 0 (no spatial dependence) to 1 (high spatial dependence). Cross‐validation was performed on semivariograms to insure their suitability for kriging. Regression coefficients, standard errors, and *r* ^2^ values of cross‐validation plots were checked before kriging. Ordinary kriging was used to interpolate values between sampling points (i.e., unmeasured locations). Ordinary kriging is a spatial interpolation technique that employs the local mean in the estimation and computes values by selecting weights to minimize estimation variance (Isaaks & Srivastava, 1989). Finally, spatial contour maps were generated using GS+ version 10.

### 2.5 Network analysis

Co‐occurrences between bacterial, archaeal, and fungal communities were assessed by performing network analysis using the maximal information coefficient (MIC) scores in the MINE statistics (Reshef et al., 2011). MIC score reveals positive, negative, and nonlinear associations among OTUs. To minimize pairwise comparisons and manage the false discovery rate (FDR; Benjamini & Hochberg, 1995), network analysis was performed only on OTUs that were present in at least two samples. This resulted in 1,006 bacterial, 105 archaeal, and 697 fungal OTUs. Relationships between microbial OTUs, vegetation types, and soil ecological processes that were significant at an FDR of 5% were then visualized in Cytoscape version 3.4.0 (Shannon et al., 2003). To indicate the most important interactions, only strong positive (*r* > 0.8), strong negative (*r* < −0.8), and strong nonlinear (MIC – *ρ* ^2^ > 0.8) relationships were shown in the networks. We compared our network against its randomized version using the Barbasi–Albert model available in *Randomnetworks* plugin in Cytoscape v2.6.1. Structural attributes of the overall network such as clustering coefficient, average degree, degree distribution, and mean shortest path were significantly different from a random network with equal number of nodes and edges. The *NetworkAnalyzer* tool was then used to calculate network topology parameters. Relationships between microbial co‐occurrences and ecological processes were visualized in Cytoscape. The OTUs with high mean degree (≥199), high closeness centrality (>0.475), and low betweenness centrality (<0.025) scores were considered as keystone taxa (Berry & Widder, 2014). Random Forest Analysis (Breiman, 2001) was used to explore the edaphic drivers of keystone taxa. The analysis was conducted using the *rfPermute* package version 2.1.5 in R (Archer, 2016). The best predictor was identified based on statistical significance with 999 permutations.

## 3 RESULTS

### 3.1 Spatial patterns of microbial abundance and potential nitrification

**Figure 1:**
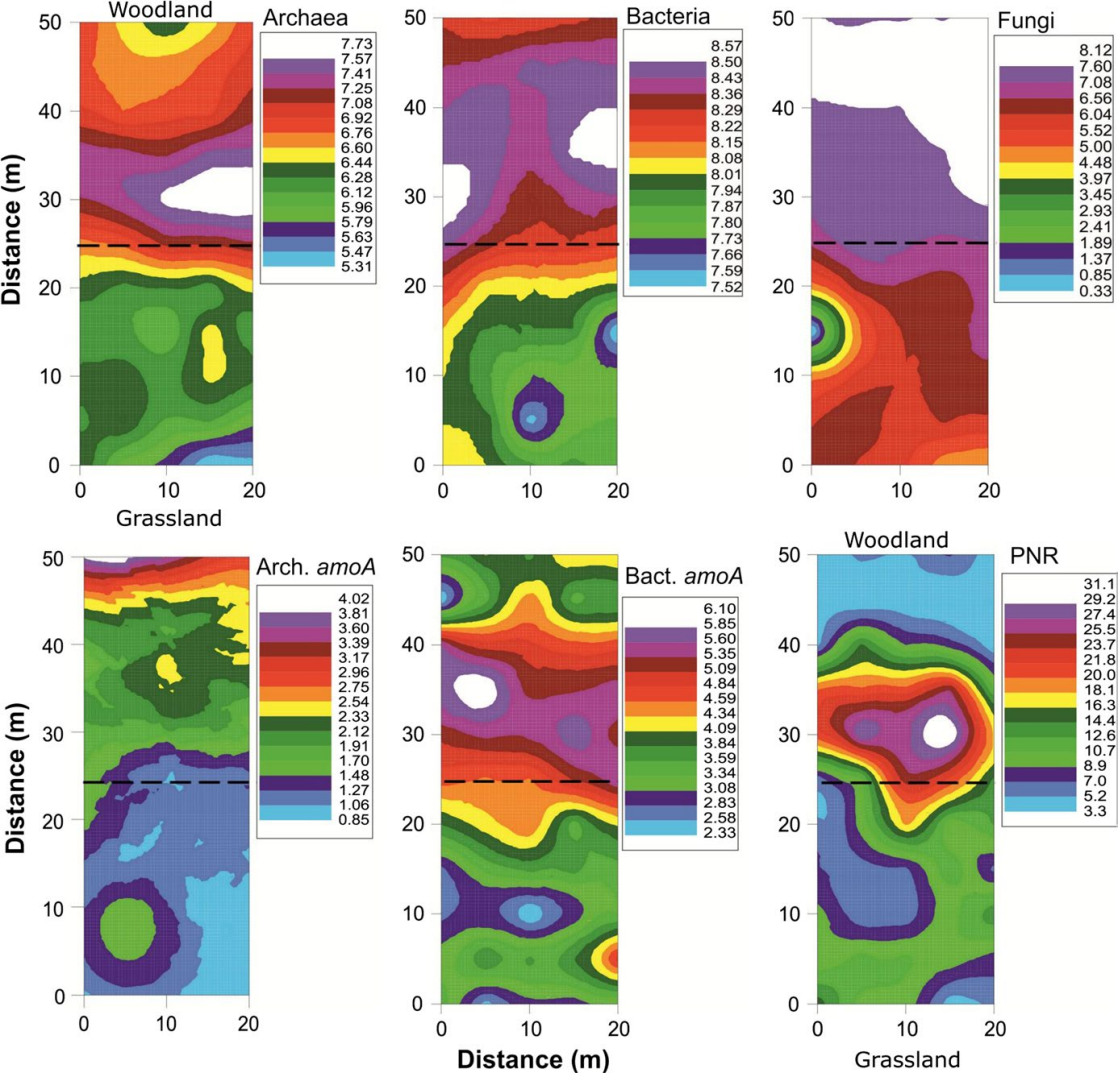
Geostatistical kriging plots showing the spatial patterns of microbial abundance and potential nitrification across the woodland‐grassland ecotone. A 50 m × 20 m grid was established across woodland and grassland in Namadgi National Park, Australia. Ordinary kriging was performed after semivariance analysis and cross‐validation. The dotted lines on kriging maps indicate the boundary between woodland in the upper half of the map and grassland in the lower half

Ordinary kriging plots revealed that the overall abundance of bacterial 16S rRNA, archaeal 16S rRNA, and fungal ITS genes were significantly (*p* < 0.05) higher in the woodland samples than in adjacent grassland samples with a distinction across the transition zone (Figure 1 upper panel; Table 1). In general, bacterial abundance was consistently high along the grid with the number of gene copies varying between 10^7^ and 10^8^ per gram of dry soil. Conversely, archaeal (10^5^–10^7^) and fungal (10^4^–10^7^) abundance showed much larger variation in copy numbers. A number of small areas were found near the transition where the abundances of all three microbial kingdoms were high. Soil PNR closely resembled the spatial patterns of bacterial ammonia oxidizers (Figure 1 lower panel). Similar to overall microbial abundance, the abundance of ammonia oxidizers was significantly (*p* < 0.05) higher in the woodland than the grassland, and a visually distinct transition zone was also noted. Bacterial *amoA* displayed a significantly (R^2^ = 0.317; *p* < 0.001) positive association with PNR across the ecotone (Supporting Information Figure S3). All five gene abundances and PNR displayed high spatial dependency (SPD = 0.631–0.999) and operated between 14 m and 36 m (Supporting Information Table S2). Spherical and Gaussian models showed significant (*R* ^2^ = 0.285–0.900; *p* < 0.01) spatial structure for all properties tested. Bacterial *amoA* and PNR structured at a smaller spatial range (14.7–23.7 m) than archaeal *amoA*, which had a spatial range >50 m.

**Table 1:** Soil microbial indices and potential nitrification rate across the woodland‐grassland ecotone at the Namadgi National Park, Australia

Soil microbiota and activities	Ecotone components		
	Woodland	Transition	Grassland
Abundance (Log copies g^−1^ dry soil)			
Bacterial 16S rRNA	8.40 (0.05)^a^	8.29 (0.08)^a^	7.84 (0.12)^b^
Archaeal 16S rRNA	6.91 (0.11)^a^	6.96 (0.21)^a^	6.10 (0.16)^b^
Fungal ITS	7.69 (0.09)^a^	7.06 (0.16)^a^	5.39 (0.66)^b^
Bacterial *amoA*	4.55 (0.25)^a^	4.82 (0.20)^a^	3.54 (0.34)^b^
Archaeal *amoA*	5.86 (0.28)^a^	5.12 (0.20)^a^	5.51 (0.19)^a^
Richness			
Archaea	42.2 (7.71)^a^	21.5 (1.91)^b^	22.1 (1.30)^b^
Bacteria	1169 (43.10)^b^	1147 (96.03)^b^	1395 (21.87)^a^
Fungi	374 (14.10)^a^	308 (27.03)^b^	337 (12.22)^ab^
Pielou's evenness			
Archaea	0.40 (0.04)^a^	0.09 (0.02)^c^	0.21 (0.03)^b^
Bacteria	0.77 (0.01)^a^	0.80 (0.01)^b^	0.82 (0.01)^b^
Fungi	0.61 (0.04)^a^	0.68 (0.04)^a^	0.72 (0.03)^a^
Diversity (Shannon‐Weaver)			
Archaea	1.45 (0.15)^a^	0.27 (0.07)^c^	0.66 (0.11)^b^
Bacteria	5.48 (0.08)^b^	5.67 (0.15)^b^	6.00 (0.03)^a^
Fungi	3.57 (0.23)^a^	3.86 (0.19)^a^	4.22 (0.21)^a^
Activity			
Nitrification potential (PNR) (μg NO_3_‐NO_2_ g^−1^ dry soil hr^−1^)	0.13 (0.04)^a^	0.36 (0.078)^b^	0.08 (0.01)^a^

*Notes*

Along the grid length (50 m), the first 20 m was woodland, 10 m was transition, and the last 20 m was grassland, resulting (*n*) in 20, 15, and 20 samples, respectively. For microbial diversity indices, *n* = 6.

Soil microbial properties were compared between woodland, transition, and grassland by performing one‐way ANOVA with Duncan post hoc test.

Different letters indicate statistical significance at *p *<* *0.05.

### 3.2 Microbial community structure, taxonomic composition, and diversity

**Figure 2:**
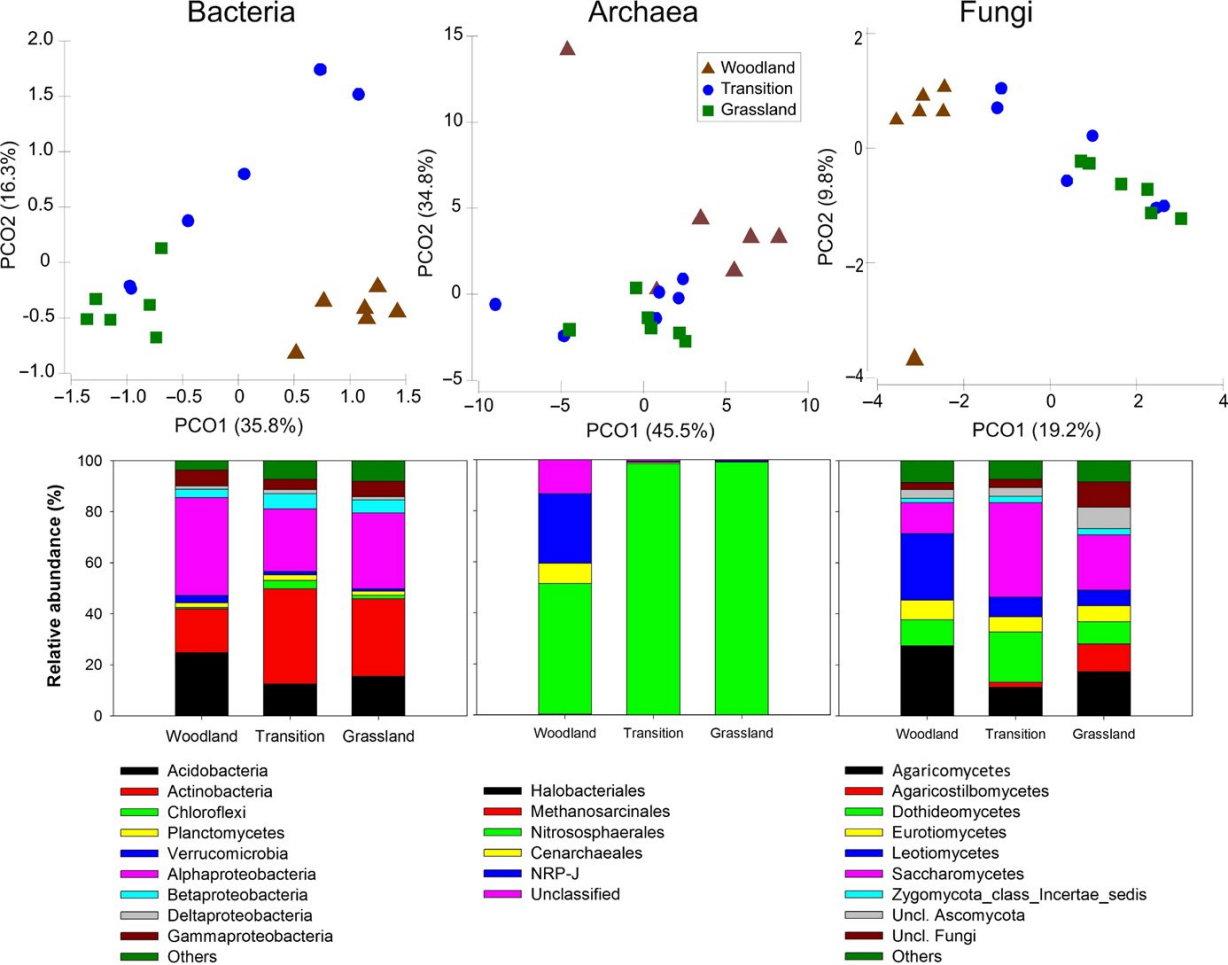
Principal coordinate analysis revealing community structure of bacteria, archaea, and fungi in woodland, grassland, and transition zone (upper panel). Stacked bar chart (bottom panel) showing relative abundance of various phyla and classes of bacteria, archaea, and fungi in woodland, grassland, and transition zone

Archaeal, bacterial, and fungal communities displayed a significant habitat effect with the woodland and grassland samples forming distinct clusters and those from the transition zone samples showing a gradient (Figure 2 upper panel). PERMANOVA confirmed such habitat effects for bacterial (Pseudo‐*F* = 4.79; *p* < 0.001), archaeal (Pseudo‐*F* = 4.42; *p* < 0.001), and fungal (Pseudo‐*F* = 3.43; *p* < 0.001) communities. Principal coordinates explained 52%, 81%, and 30% variation in bacterial, archaeal, and fungal communities, respectively. Similar to community structure, taxonomic composition was also influenced by the habitat edge (Figure 2 lower panel). For bacteria, *Acidobacteria*,* Actinobacteria,* and *Alphaproteobacteria* were the dominant members, representing more than 80% of total bacterial abundance across the ecotone. *Acidobacteria* and *Alphaproteobacteria* were more abundant in the woodland samples while *Betaproteobacteria* and *Chloroflexi* were the most common within the transition zone (*p* < 0.05). *Nitrososphaerales* was the predominant archaeal group, especially, in the grassland and transition zone samples where it represented up to 99% of the total archaea. On the other hand, a number of fungal groups showed significant (*p* < 0.01) differences across the ecotone. For example, *Agaricomycetes* and *Leotiomycetes* were 2–3 times more abundant in woodland samples than either the transition zone or grassland samples. Similarly, *Saccharomycetes* and *Dothideomycetes* were significantly more abundant (*p* < 0.05) in the transition zone and *Agaricostilbomycetes* (*p* < 0.001) in the grassland. Microbial alpha diversity indices showed contrasting patterns across the ecotone (Table 1). For example, richness, evenness, and diversity of bacteria were significantly (*p* < 0.05) lower in the woodland samples than the transition zone and grassland. Conversely, archaeal richness, evenness, and diversity were significantly (*p* < 0.05) higher in the woodland than either the transition zone or the grassland samples. This indicates that the woodland samples harbored a less diverse bacterial community and a highly diverse archaeal community. On the other hand, the relatively N‐ and P‐rich grassland samples supported a diverse bacterial community (Supporting Information Table S1). Fungi also had a significantly higher richness in woodland samples.

### 3.3 Microbial co‐occurrences

**Figure 3:**
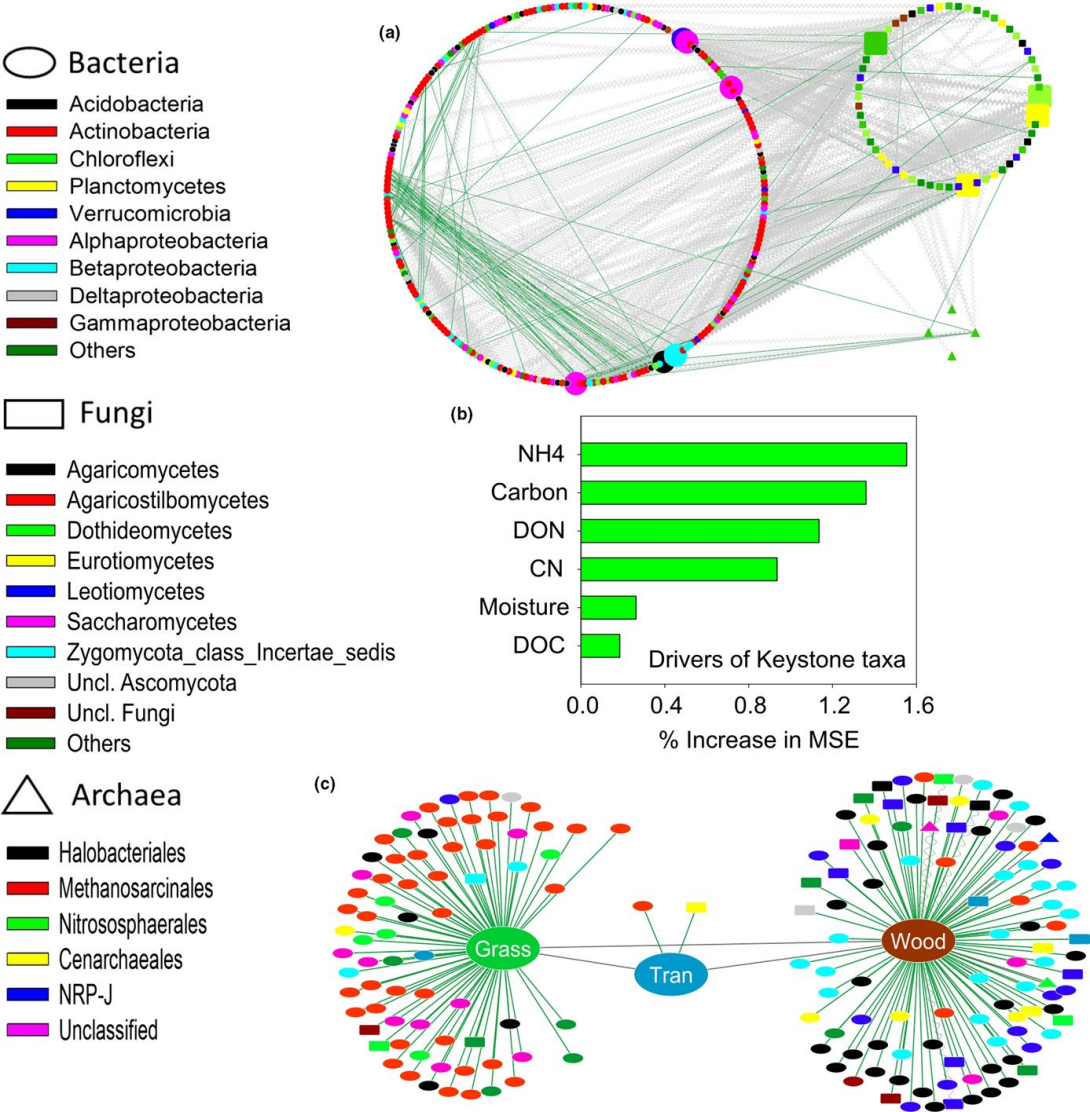
(a) Microbial network showing co‐occurrences of bacterial, archaeal, and fungal OTUs. This network of top 1,000 interactions consisted of 324 nodes. Enlarged nodes represent the top ten microbial keystone taxa of which six were bacterial and four fungal. (b) Results of Random Forest Analysis showing the edaphic drivers of microbial keystone taxa. The MSE indicates vector of mean square errors. (c) Microbial co‐occurrences in the woodland, grassland, and transition zone. This network comprised 193 nodes. To indicate the most important interactions, only strong positive (*r* > 0.8), strong negative (*r* < −0.8), and strong nonlinear (MIC – *ρ* ^2^ > 0.8) relationships were shown in the networks. Oval nodes represent bacterial OTUs, rectangular nodes represent fungal OTUs, and triangular nodes represent archaeal OTUs. Color of the nodes represents different taxonomic groups while green, red, and wavy lines represent positive, negative, and nonlinear relationships, respectively. Only statistically significant (*p* < 0.05) relationships are shown

**Figure 4:**
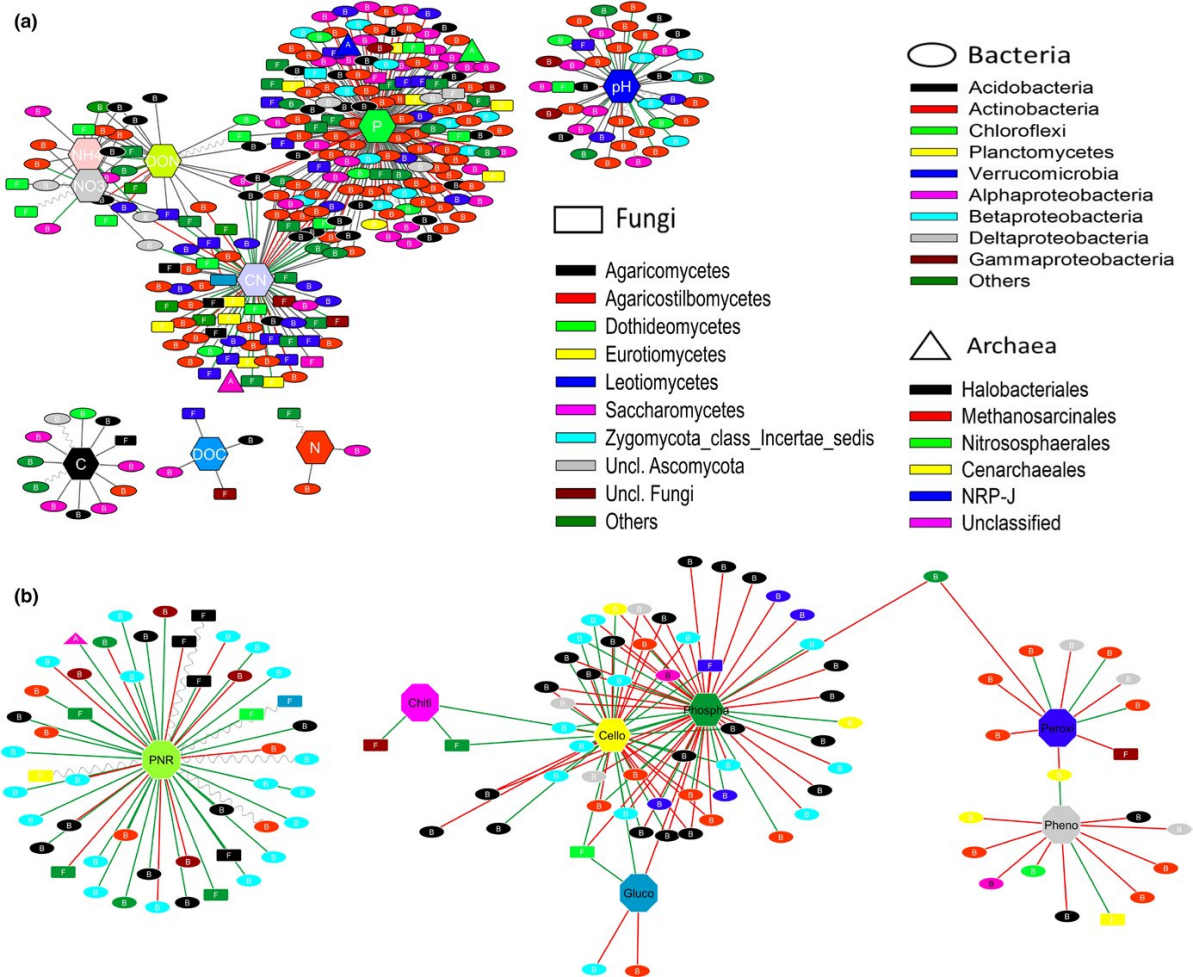
Relationship among microbial co‐occurrence, soil properties, and ecological processes. (a) Archaeal, bacterial, and fungal OTUs formed distinct clusters with soil chemical properties. Large clusters such as P consisted of 164 nodes, C:N comprised 76 nodes, and pH consisted of 42 nodes. (b) Clusters of microbial OTUs linked to potential nitrification and extracellular enzyme activities. The cluster of PNR comprised 49 nodes. To indicate the most important interactions, only strong positive (*r* > 0.8), strong negative (*r* < −0.8), and strong nonlinear (MIC – *ρ* ^2^ > 0.8) relationships were shown in the networks. Oval nodes represent bacterial OTUs, rectangular nodes represent fungal OTUs, and triangular nodes represent archaeal OTUs. Color of the nodes represents different taxonomic groups while green, red, and wavy lines represent positive, negative, and nonlinear relationships, respectively. Only statistically significant (*p* < 0.05) relationships are shown

Network analysis showed distinct co‐occurrences of archaeal, bacterial, and fungal members across the woodland, grassland, and transition zone (Figure 3). The microbial network including the top 1000 MIC scores comprised 324 nodes (260 bacterial, four archaeal, and 60 fungal OTUs; Figure 4). Among the top 10 keystone taxa, six were bacteria and four were fungi (Table 2; Figure 3a). Half of the bacterial keystone taxa belonged to the class *Alphaproteobacteria* whereas most fungal keystone taxa were members of the *Ascomycetes*. Two bacterial OTUs belonged to *Rhizobiales* and *Burkholderiales*. Random Forest Analysis showed that ammonium, total carbon, and C:N ratio were the major determinants of the abundance of microbial keystone taxa (Figure 3b). The network across the ecotone consisted of 193 nodes of which woodland and grassland were associated with 116 and 74 nodes, and the transition zone connected to two nodes (Figure 3c). The overall network comprised 1767 nodes and had a diameter of 6 and a radius of 4 (Supporting Information Figure S3). Woodland and grassland formed clusters away from the transition zone. While the network was dominated by bacteria, fungal and archaeal nodes were also abundant in the woodland. Overall, microbial and co‐occurrences were considerably different in three habitat components across the ecotone.

**Table 2:** Network features and taxonomy of top ten keystone taxa. OTUs with highest degree, highest closeness centrality, and lowest betweenness centrality were selected as the keystone taxa

OTUid	Network features			Taxonomy		
	Betweenness centrality	Closeness centrality	Degree	Kingdom	Phylum or class	Order
Botu781	0.024	0.502	265	Bacteria	*Acidobacteria*	*Acidobacteriales*
Fotu671	0.021	0.510	254	Fungi	*Eurotiomycetes*	*Chaetothyriales*
Fotu695	0.026	0.508	250	Fungi	*Dothideomycetes*	*Capnodiales*
Botu706	0.015	0.499	246	Bacteria	*Alphaproteobacteria*	*Rhodospirillales*
Fotu626	0.013	0.502	241	Fungi	*Zygomycota*	*Mortierellales*
Botu914	0.012	0.501	238	Bacteria	*Verrucomicrobia*	*Pedosphaerales*
Botu257	0.008	0.484	227	Bacteria	*Alphaproteobacteria*	*Caulobacterales*
Botu890	0.015	0.494	220	Bacteria	*Alphaproteobacteria*	*Rhizobiales*
Fotu569	0.011	0.486	210	Fungi	*Eurotiomycetes*	*Chaetothyriales*
Botu81	0.005	0.476	199	Bacteria	*Betaproteobacteria*	*Burkholderiales*

### 3.4 Relationships among microbial co‐occurrences, soil properties, and ecological processes

Microbial co‐abundances were linked to soil properties and ecological processes, with the subnetworks comprising mainly bacterial OTUs (Figure 4; Supporting Information Figure S4). For soil properties, total soil P formed the cluster with maximum nodes followed by C:N ratio and pH (Figure 4a). Mineral N, DON, C:N, and P clusters shared several nodes and were predominantly connected through positive and linear edges. In general, the subnetworks were dominated by *Alphaproteobacteria* and *Actinobacteria* OTUs in bacteria. For soil ecological processes, PNR formed a large and distinct cluster away from soil enzymes and predominantly consisted of bacterial nodes but no archaeal nodes, whereas soil enzymes formed individual clusters but were interconnected through shared nodes (Figure 4b). This is especially true for cellobiohydrolase and phosphatase activities that had a large shared guild. β‐glucosidase had the smallest cluster but was connected with both cellobiohydrolase and phosphatase through a fungal node belonging to *Dothideomycetes*. Similar to soil P content, phosphatase formed the largest clusters. These clusters of soil enzymes were also dominated by bacterial OTUs. Potential nitrification showed consistently significant (*p* < 0.01) correlations with soil properties and extracellular enzymes (Supporting Information Table S3). Bacterial *amoA* gene copy number had a strong association with PNR activity in these relatively N‐rich soils, and this was also supported by the fact that bacterial *amoA* was strongly (*p* < 0.01) correlated with soil N content. Taken together, microbial co‐occurrences reflected the differences in soil properties and ecological processes.

## 4 DISCUSSION

### 4.1 Microbial communities across the ecotone

In this study, we explored the abundance, structure, and co‐occurrences of soil archaea, bacteria, and fungi, and their relationships with relevant soil ecological processes along a woodland‐grassland ecotone. Firstly, using spatial interpolation, we showed how the overall abundance of archaea, bacteria, and fungi changed between woodland and grassland soil samples. The woodland samples had significantly higher microbial abundance than was observed for the grassland samples with a visually distinct transition zone. This higher abundance was observed generally for overall archaea, bacteria, and fungi, and specifically for ammonia oxidizing archaea and bacteria. The overall gene copy numbers of these microbial groups we found in our woodland and grassland soils are comparable to previous studies (Banerjee, Baah‐Acheamfour et al., 2016; Gleeson et al., 2010; Kemnitz, Kolb, & Conrad, 2007; Lauber, Strickland, Bradford, & Fierer, 2008). Secondly, our analyses of α‐ diversity indices showed that the woodland samples had significantly higher diversity of archaea while the grassland samples had significantly higher diversity of bacteria. It should be noted that the grassland soils at our site had significantly higher N and P levels than the woodland soils. Typically, bacteria are more responsive to nutrient‐rich conditions than archaea (Carey, Dove, Beman, Hart, & Aronson, 2016), which indicates their copiotrophic nature (Fierer, Bradford, & Jackson, 2007).

Our results show that the habitat edge between woodland grassland significantly influenced microbial β‐diversity. Microbial communities formed distinct clusters in woodland and grassland samples with the transition zone forming a gradient between those two adjoining systems. Moreover, OTUs belonging to *Acidobacteria* in bacteria and *Agaricomycetes* and *Leotiomycetes* in fungi was significantly higher in the woodland samples than elsewhere. Several members of these oligotrophic microbial groups are involved in wood decomposition, and our results are consistent with previous studies reporting greater abundance of these groups in forest soils (Edwards & Zak, 2011; Jones et al., 2009). Several members of the *Agaricomycetes* are ectomycorrhizal (van der Heijden, Martin, Selosse, & Sanders, 2015), which may also explain their higher abundance in the woodland soils. Similarly, we found that the number of OTUs belonging to *Agaricostilbomycetes* was significantly higher in the grassland samples than the woodland samples. Interestingly, a previous study showed a positive relationship between the abundance of OTUs of this group and plant community richness in grassland (LeBlanc, Kinkel, & Kistler, 2014). Overall, microbial diversity and composition were significantly influenced by the habitat edge as revealed across this ecotone.

### 4.2 Potential nitrification driven by bacterial ammonia oxidizers

Bacterial rather than archaeal ammonia oxidizers drove potential nitrification in the N‐rich soils at this site, and this pattern was consistently shown by multiple analytical approaches employed in this study. For example, ordinary kriging revealed that both bacterial *amoA* and PNR had visually similar spatial patterns and operated at similar spatial ranges. Consequently, these groups were also positively correlated (*p* < 0.001) across the ecotone. Network analysis further indicated that the PNR subnetwork was dominated by unclassified members of *Betaproteobacteria* and not archaea. It should be noted that soils across this ecotone were relatively N rich with average %N, NH_4_‐N and DON of 0.287% (w/w), 12.1 μg and 78.8 μg per gram of soil, respectively (Banerjee, Bora et al., 2016). While archaeal ammonia oxidizers are important for nitrification and dominant in many ecosystems (Leininger et al., 2006), they are well‐acknowledged for their oligotrophic nature (Erguder, Boon, Wittebolle, Marzorati, & Verstraete, 2009; Hatzenpichler, 2012). On the other hand, bacterial ammonia oxidizers are typically copiotrophic which makes them particularly suited for more nutrient rich soils. Interestingly, archaeal *amoA* was more abundant than bacterial *amoA* in both woodland and grassland soils in our study, but despite this, bacterial ammonia oxidizers displayed a stronger correlation with potential nitrification. Previous studies have similarly found that archaeal ammonia oxidizers are less responsive to nitrification in N‐rich soils even when they are relatively more abundant than their bacterial counterparts (Di et al., 2009). The higher responsiveness of ammonia oxidizing bacteria in N‐rich soils was also noted in a recent global meta‐analysis (Carey et al., 2016). The different spatial ranges of bacterial and archaeal ammonia oxidizers in our study indicate a possible niche differentiation of these communities as previously suggested (Prosser & Nicol, 2012). While the majority of *Betaproteobacteria* nodes in the PNR subnetwork were unclassified members, the association between the PNR subnetwork and *Betaproteobacteria* members reinforces the importance of this bacterial group for nitrification in N‐rich soils.

### 4.3 Microbial co‐occurrences across ecotone

We found a similarity between microbial co‐occurrence and spatial patterns. For example, microbial nodes in the woodland, grassland, and transition zone were structured into separate clusters with the woodland habitat having a significantly higher number of nodes. Similarly, kriging showed a significantly higher abundance of all microbial groups in the woodland samples. Importantly, our results illustrate how network complexity, indicated by the number of nodes and edges, changes between two adjoining ecological systems within one landscape and how archaeal, fungal, and bacterial patterns of co‐occurrence are influenced in the transition zone. Previous studies using network analysis have often only assessed bacterial communities and not fungal or archaeal communities (Banerjee, Baah‐Acheamfour et al., 2016; Barberán et al., 2012; Shi et al., 2016; Vick‐Majors et al., 2014). Thus, the roles of these latter groups have been underrepresented in microbial network analyses (Ma et al., 2016; Steele et al., 2011). While the networks were dominated by bacterial nodes, fungal and archaeal nodes were also abundant. Our results highlight the importance of assessing interkingdom associations in soil microbial networks.

### 4.4 Relationships between microbial co‐occurrences and ecological processes

Linking microbial community composition to function is a central goal in ecology (Graham et al., 2016; Prosser et al., 2007). In this study, soil P and C:N formed large clusters dominated by bacterial nodes and these clusters were also connected with other C and N properties. Similarly, the processes of C, N, and P cycling were also correlated with microbial co‐occurrence. Interestingly, soil P and phosphatase activity formed the largest clusters in their respective networks whereas two lignolytic enzymes (phenol oxidase and peroxidase) formed joined clusters. Extracellular enzymes are involved in the decomposition and mineralization of soil organic matter, which is a “broad” process that involves many steps and operated by functionally and taxonomically diverse generalist microbial groups (Fierer et al., 2007; Schimel & Schaeffer, 2012). On the other hand, ammonia oxidation is a “narrow” process facilitated by specialist groups of bacteria and archaea (Kowalchuk & Stephen, 2001). The distinct cluster of PNR was mainly dominated by *Betaproteobacteria*, reinforcing the observation that nitrification at this site was driven by ammonia oxidizing bacteria.

### 4.5 Keystone taxa and determinants

A useful feature of network analysis is that it can identify “hubs” or keystone taxa that have significant influence on the structure and functioning of microbiomes (Newman, 2003). Identifying keystone taxa and the factors that drive their abundance and spatiotemporal distribution is of particular importance in microbial ecology. The concept of keystone taxa was originally proposed some decades ago by ecologist Paine (1966). Keystone taxa have been identified in microbial communities both statistically (Banerjee, Kirkby et al., 2016; Hartman et al., 2018; Lupatini et al., 2014; Shi et al., 2016) and empirically (Curtis et al., 2014; Fisher & Mehta, 2014; Hajishengallis, Darveau, & Curtis, 2012). Berry and Widder (2014) used network‐based scores such as high mean degree, high closeness centrality, and low betweenness centrality to identify keystone taxa with 85% accuracy. Using the method proposed by Berry and Widder (2014), we identified six bacterial and four fungal OTUs in this study as representing the top ten keystone taxa. In a recent study, we also reported that bacterial and fungal keystones were significantly correlated to organic matter decomposition in an agricultural soil (Banerjee, Kirkby et al., 2016). Soil carbon and nitrogen contents likewise emerged as the drivers of keystone taxa that we identified here across the woodland‐grassland ecotone. One limitation of this study is that high‐throughput sequencing of microbial communities was performed on 18 soil samples. These samples were randomly selected equally from the three zones with six samples at each habitat component. Thus, careful consideration was made to obtain representative samples across this woodland‐grassland ecotone. Moreover, the selection of a single ecotone site in this study was based our previous observation that spatial patterns of a range of soil properties and extracellular enzyme activities were similar across two native woodland‐grassland ecotones (including this one) located approximately 150 km apart (Banerjee, Bora et al., 2016).

## 5 CONCLUSION

Using geostatistics, quantitative PCR, high‐throughput sequencing and network analysis, we demonstrated spatial patterns and co‐occurrences of archaeal, bacterial, and fungal communities across a woodland‐grassland ecotone. The abundance, structure, and taxonomic composition of soil microbial communities were significantly different in the transition zone than the woodland and grassland. Microbial networks predominantly comprised positive interactions that reflected the high C, N, and P levels at this site. Microbial co‐occurrences showed clusters based on habitats, soil properties, and ecological processes. Although microbial networks were dominated by bacterial OTUs, fungal and archaeal members were also abundant, highlighting the importance of interkingdom associations in soil microbial networks. Nitrification was driven by ammonia‐oxidizing bacteria, and this was supported by the dominance of *Betaproteobacteria* OTUs in the PNR subnetwork. A coherence of spatial patterns and co‐occurrences of microbial communities was thus demonstrated across the ecotone.

## CONFLICT OF INTEREST

None declared.

## AUTHOR CONTRIBUTIONS

S.B. designed the study and performed analyses. S.B., A.E.R., and P.H.T. conducted the soil sampling and made substantial contributions to the writing. A.B. assisted in data analyses, and M.v.d.H. contributed to the writing of the article.

## DATA ACCESSIBILITY

The raw sequence data of bacterial 16S rRNA, archaeal 16S rRNA, and fungal ITS are available under the NCBI BioProject Accession number PRJNA427915 (SRA accession SRP 131862).
