# Supplementary figures and images for: Linking microbial co‐occurrences to soil ecological processes across a woodland‐grassland ecotone
Source: Ecol Evol. 2018 Jul 22;8(16):8217–30. doi: 10.1002/ece3.4346 (PMC6145019; doi:10.1002/ece3.4346)

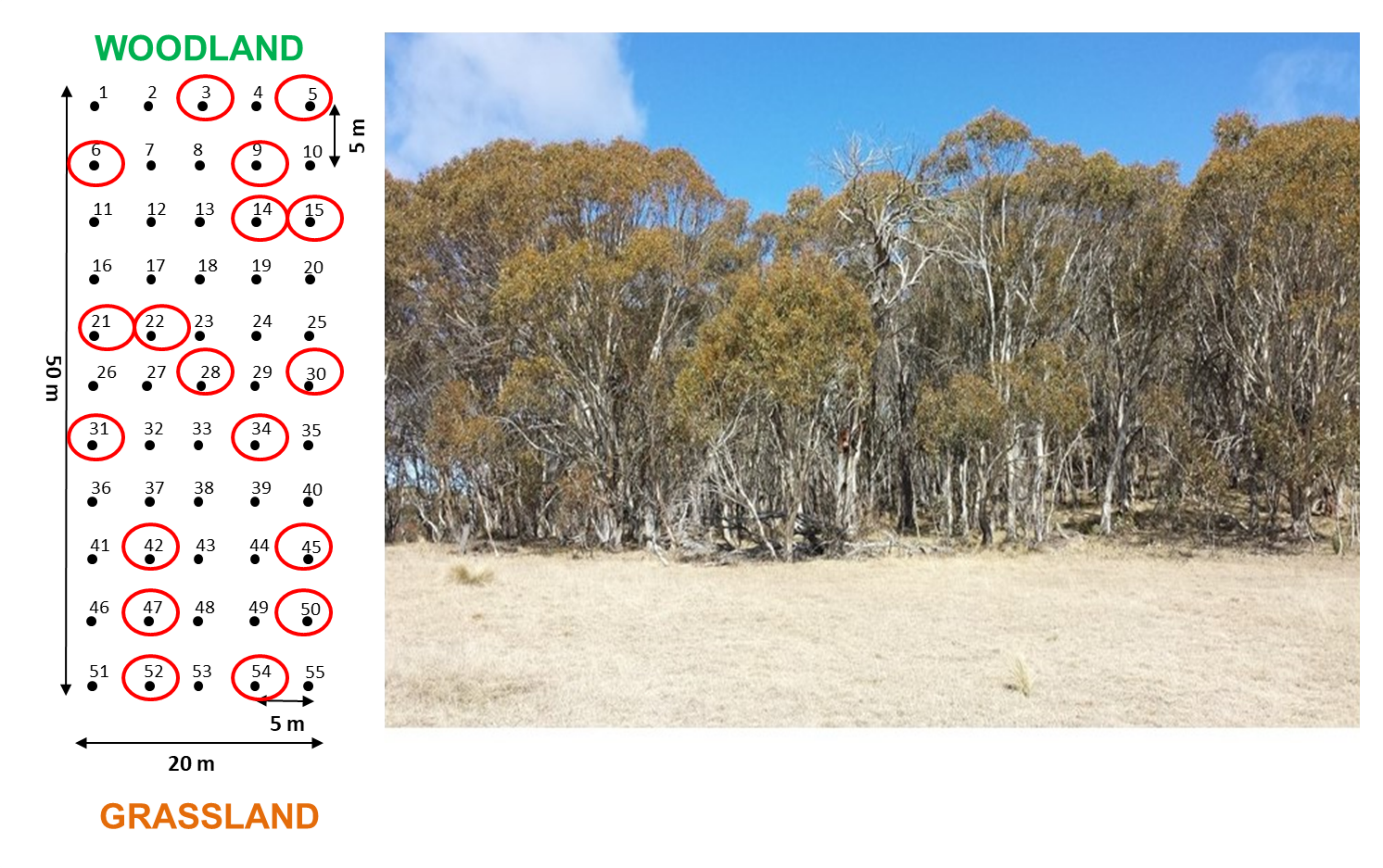

Supplement: Supplementary file 1 [file ECE3-8-8217-s001.png]

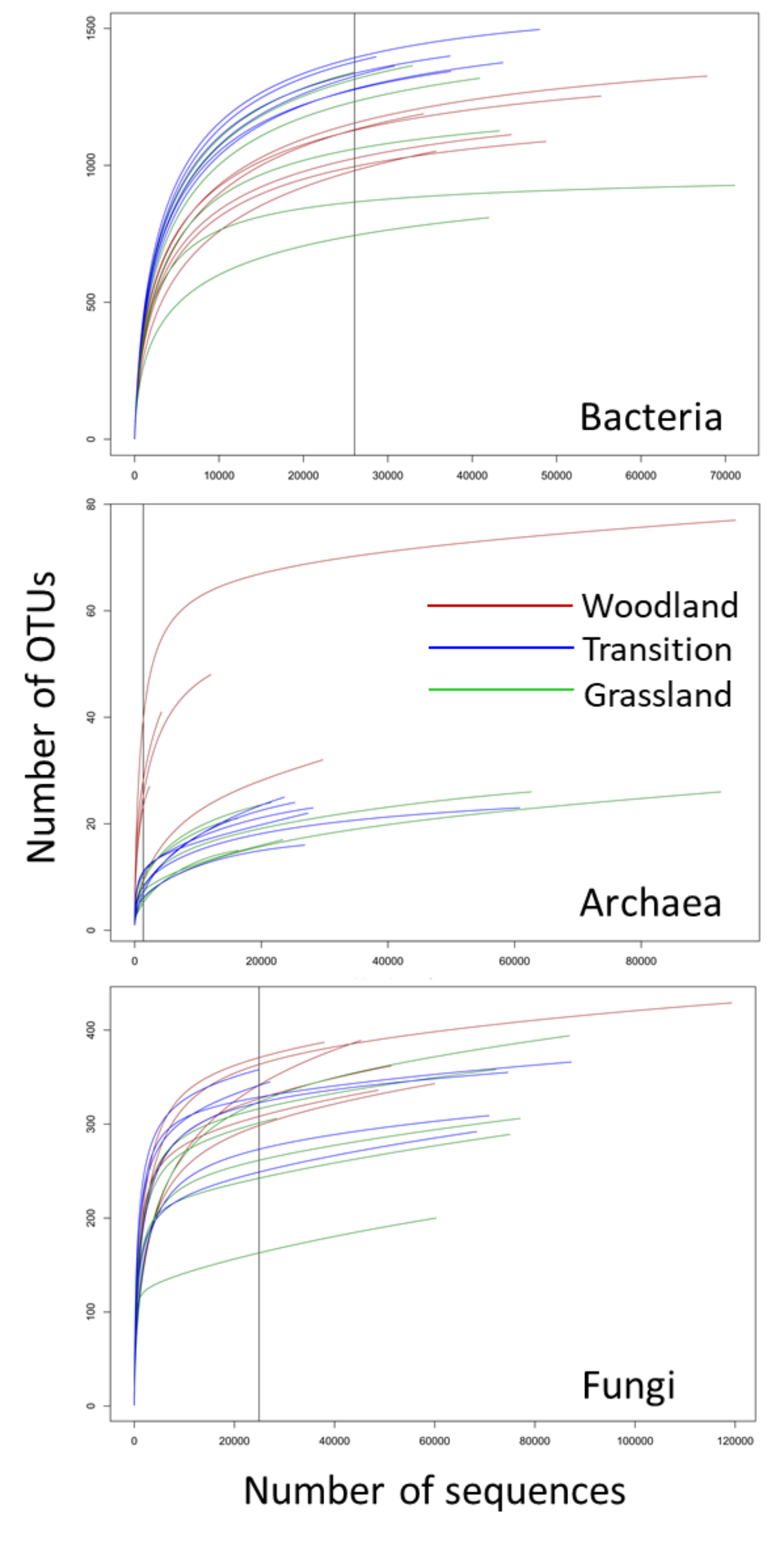

Supplement: Supplementary file 2 [file ECE3-8-8217-s002.png]

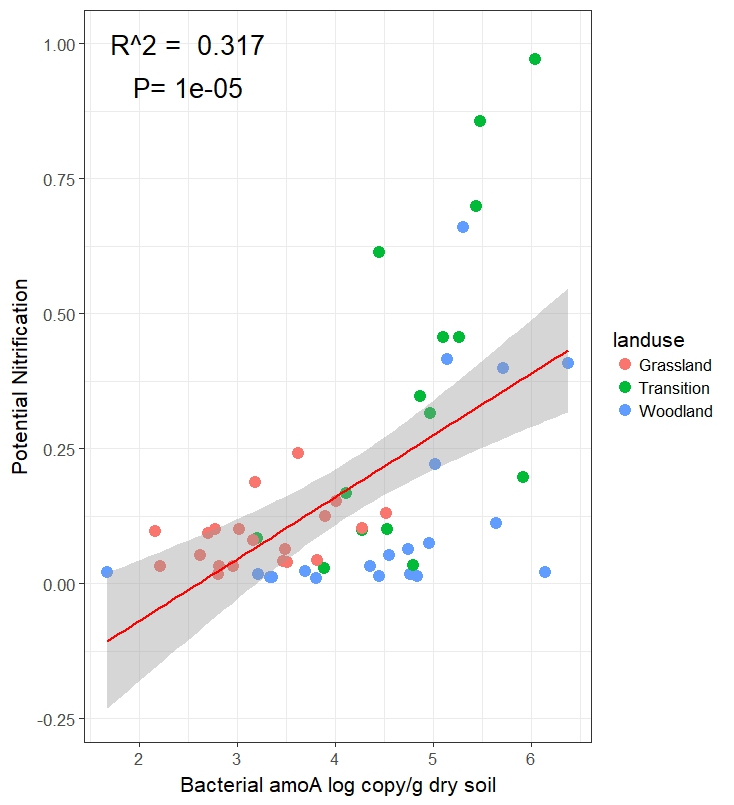

Supplement: Supplementary file 3 [file ECE3-8-8217-s003.jpeg]

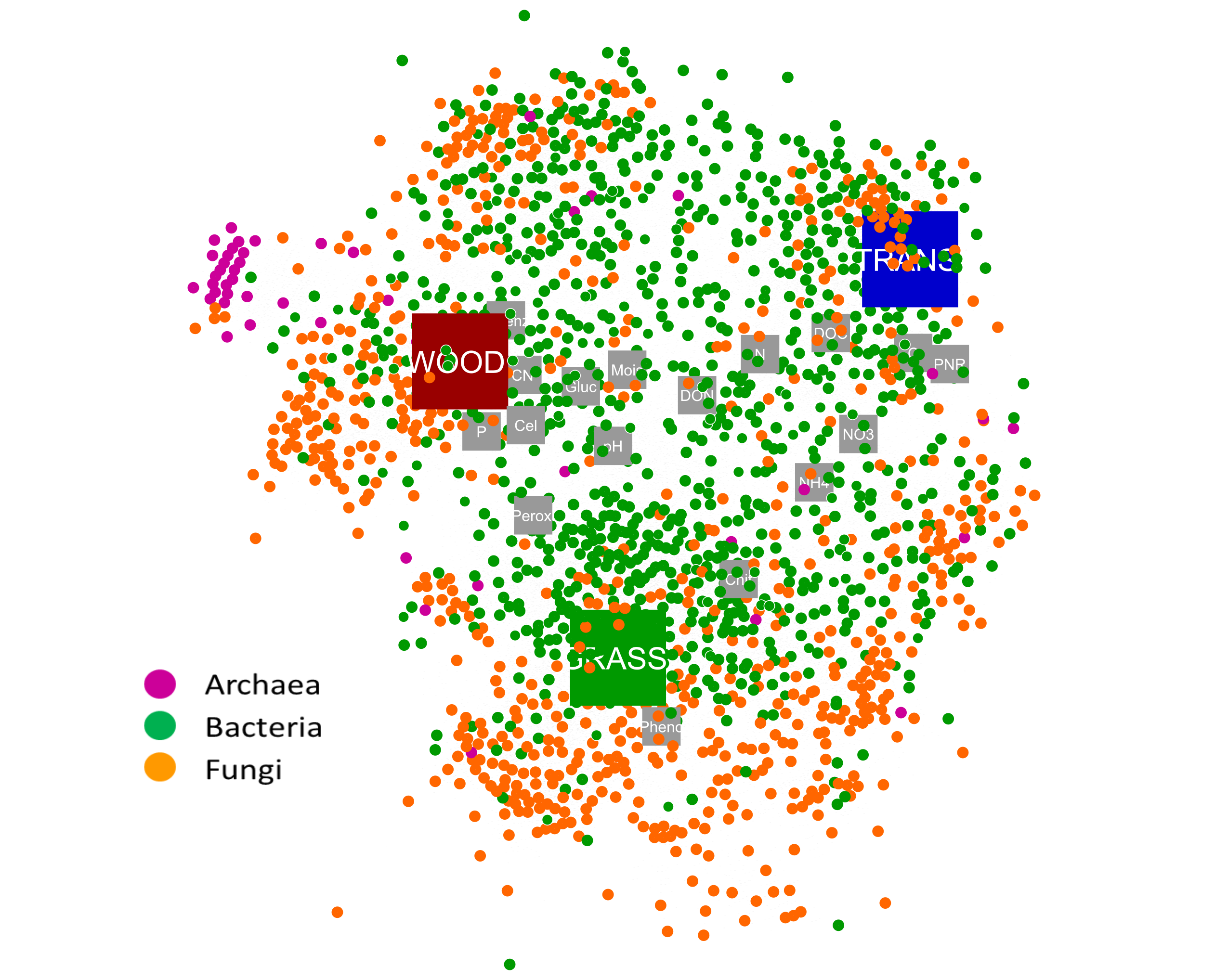

Supplement: Supplementary file 4 [file ECE3-8-8217-s004.png]
