# Supplementary material for: Linking microbial co‐occurrences to soil ecological processes across a woodland‐grassland ecotone
Source: Ecol Evol. 2018 Jul 22;8(16):8217–30. doi: 10.1002/ece3.4346 (PMC6145019; doi:10.1002/ece3.4346)
Supplement: Supplementary file 5 [file ECE3-8-8217-s005.docx]

**qPCR conditions and quality assessment**

Each 10 μl reaction contained 3 μl of DNA template, 6 μl of Qiagen Quantitech SYBR Green Mastermix, 0.45 μl of 10 pmol forward and reverse primers, and 0.1 μl of BSA. Thermal cycling conditions were 1 cycle of 15 min at 95°C; 40 cycles of 95°C for 15 s, annealing, 72°C for 60 s, 80°C for 15 s; and 1 cycle of 95°C for 15 s, 60 to 95°C. For bacterial 16S rRNA and *amoA*, the annealing temperatures were 60°C for 45 s and 1 min, respectively. Archaeal 16S rRNA and *amoA* had annealing temperatures of 54°C for 30 s and 55°C for 1 min, respectively. Fungal ITS had an annealing temperature of 56°C for 30 s. Standard curves for bacterial 16S rRNA and fungal ITS were prepared using purified PCR products obtained from *Bradyrhizobium* sp and *Nigrospora* sp, respectively. Standards for bacterial *amoA*, archaeal 16S rRNA and *amoA* genes were prepared from cloned plasmids. Standard curves linear over five orders of magnitude and *r*^2^ value of 0.99 or higher were selected. The efficiency of the reaction was between 80% and 94% (based on the slopes of the standard curves). All samples and standards were run with four replicates. Consistency of Y-intercept values was checked between different qPCR runs and the specificity of the amplified products was assessed by melting curve analysis.

**Table S1.** Soil properties and extracellular enzyme activities across the ecotone at the Namadgi National Park, Australia.

| **Soil properties and enzyme activities** | **Ecotone components** | | |
| --- | --- | --- | --- |
|  | *Woodland* | *Transition* | *Grassland* |
| *Soil properties* |  |  |  |
| Gravimetric moisture | 29.3 (1.05) ^a^* | 33.5 (1.48) ^b^ | 31.6 (0.47) ^ab^ |
| pH | 4.57 (0.98) ^a^ | 4.71 (0.10) ^a^ | 4.79 (0.34) ^a^ |
| Total carbon, % (w/w) | 5.10 (0.52) ^b^ | 5.43 (0.77) ^b^ | 3.52 (0.15) ^a^ |
| Total nitrogen, % (w/w) | 0.287 (0.03) ^a^ | 0.390 (0.04) ^b^ | 0.266 (0.00) ^a^ |
| C/N ratio | 18.2 (0.36) ^b^ | 13.6 (0.32) ^a^ | 13.2 (0.21) ^a^ |
| NH_4_-N, μg g^-1^ soil | 12.1 (1.99) ^ab^ | 16.4 (2.23) ^b^ | 10.9 (0.65) ^a^ |
| NO_3_-N, μg g^-1^ soil | 6.52 (4.73) ^a^ | 4.35 (1.32) ^a^ | 1.01 (0.47) ^a^ |
| Dissolved organic carbon, μg g^-1^ soil | 442.0 (68.9) ^a^ | 461.1 (38.1) ^a^ | 360.5 (13.7) ^a^ |
| Dissolved organic nitrogen, μg g^-1^ soil | 76.8 (10.4) ^a^ | 92.6 (5.50) ^a^ | 78.8 (3.47) ^a^ |
| Total P, μg g^-1^ soil | 515.6 (28.4) ^a^ | 773.1 (26.5) ^b^ | 799.8 (41.1) ^b^ |
| Inorganic P, μg g^-1^ soil | 108.1 (6.85) ^a^ | 173.5 (13.1) ^b^ | 152.3 (11.9) ^b^ |
| Organic P, μg g^-1^ soil | 407.4 (23.7) ^a^ | 599.6 (19.0) ^b^ | 647.5 (33.8) ^b^ |
|  |  |  |  |
| *Extracellular enzyme activities* |  |  |  |
| Cellobiohydrolase, nmol h^-1^ g^-1^ dry soil | 60.6 (10.4) ^a^ | 71.9 (3.81) ^a^ | 67.6 (3.89) ^a^ |
| Chitinase, nmol h^-1^ g^-1^ dry soil | 18.8 (4.61) ^a^ | 14.3 (1.06) ^a^ | 13.5 (1.25) ^a^ |
| Glucosidase, nmol h^-1^ g^-1^ dry soil | 11.6 (3.32) ^a^ | 7.58 (0.40) ^a^ | 7.40 (0.50) ^a^ |
| Phosphatase, nmol h^-1^ g^-1^ dry soil | 1.57 (0.06) ^a^ | 1.66 (0.02) ^a^ | 1.62 (0.02) ^a^ |
| Phenol oxidase, nM DOPA h^-1^ g^-1^ dry soil | 40.4 (5.90) ^a^ | 31.9 (4.65) ^a^ | 79.2 (6.78) ^b^ |
| Peroxidase, nM DOPA h^-1^ g^-1^ dry soil | 35.9 (4.72) ^a^ | 47.1 (6.20) ^a^ | 67.8 (5.16) ^b^ |

Along the grid length (50 m), the first 20 m was woodland, 10 m was transition and the last 20 m was grassland, resulting (n) in 20, 15 and 20 samples, respectively.

# Soil properties and enzyme activities were compared between woodland, transition and grassland at each site by performing one-way ANOVA with Duncan *post hoc* test.

*different letters suggest statistical significance at P<0.05

**Table S2.** Spatial properties of potential nitrification and the abundances of overall microbial communities and ammonia oxidizers.

| Variable | SPD^#^ | R^2^ | Range | Model |
| --- | --- | --- | --- | --- |
| Bacterial 16S rRNA | 0.631 | 0.693 | 36.4 | Spherical |
| Fungal ITS | 0.704 | 0.309 | 24.4 | Spherical |
| Archaeal 16S rRNA | 0.711 | 0.632 | 32.0 | Spherical |
| Bacterial *amoA* | 0.906 | 0.285 | 23.7 | Spherical |
| Archaeal *amoA* | 0.803 | 0.900 | >50 m | Gaussian |
| Potential nitrification | 0.999 | 0.826 | 14.7 | Gaussian |

#SPD indicates spatial dependency calculated as SPD=C / (C + C_0_), where C is the structural variance, C_0_ is the nugget, and C+C_0_ is the sill. Values of SPD vary from 0 (no spatial dependence) to 1 (high spatial dependence).

**Table S3.** Pearson correlation coefficients among microbial abundances, potential nitrification, extracellular enzymes and soil properties.

|  | PNR | Bacterial *amoA* | Archaeal *amoA* | Bacterial 16S | Archaeal 16S | Fungal ITS |
| --- | --- | --- | --- | --- | --- | --- |
| *Soil properties* |  |  |  |  |  |  |
| Moisture | 0.524** | ns | ns | ns | ns | ns |
| pH | -0.289* | ns | ns | ns | -0.396** | ns |
| NH4 | 0.320* | ns | ns | ns | ns | ns |
| NO3 | 0.354* | 0.307* | ns | ns | 0.281* | ns |
| Total C | 0.754** | 0.435** | ns | 0.262* | 0.580** | ns |
| Total N | 0.805** | 0.389** | ns | ns | 0.519** | ns |
| C:N | ns | ns | ns | 0.430** | 0.302* | 0.439** |
| DOC | 0.582** | 0.337** | ns | ns | 0.351* | ns |
| DON | 0.574** | ns | ns | ns | 0.294* | ns |
| Total P | 0.287* | ns | ns | ns | ns | ns |
| Inorganic P | 0.464** | ns | ns | ns | 0.335* | ns |
| Organic P | ns | ns | ns | ns | -ns | ns |
|  |  |  |  |  |  |  |
| *Extracellular enzymes* |  |  |  |  |  |  |
| Cellobiohydrolase | 0.342** | ns | ns | ns | ns | ns |
| Chitinase | ns | ns | ns | ns | ns | ns |
| Phosphatase | 0.390** | ns | ns | ns | ns | ns |
| Phenol Oxidase | -0.421** | -0.377** | ns | -0.451** | -0.461** | ns |
| Peroxidase | ns | -0.343** | ns | -0.375* | -0.312* | ns |
|  |  |  |  |  |  |  |
| *Microbial properties* |  |  |  |  |  |  |
| Bacterial *amoA* | 0.433** | na | ns | 0.290* | 0.435** | 0.332* |
| Archaeal *amoA* | ns | ns | na | ns | ns | ns |
| Bacterial 16S | ns | 0.290* | ns | na | 0.520** | 0.288* |
| Archaeal 16S | 0.586** | 0.435** | ns | 0.520** | na | 0.426** |

*and ** indicate statistical significance at P<0.05 and P<0.01, respectively

ns indicates non-significant correlation

na indicates not applicable

No correlations found with Glucosidase activity
